# Greenhouse Gas Flux and Crop Productivity after 10 Years of Reduced and No Tillage in a Wheat-Maize Cropping System

**DOI:** 10.1371/journal.pone.0073450

**Published:** 2013-09-03

**Authors:** Shenzhong Tian, Yu Wang, Tangyuan Ning, Hongxiang Zhao, Bingwen Wang, Na Li, Zengjia Li, Shuyun Chi

**Affiliations:** 1 State Key Laboratory of Crop Biology, Shandong Key Laboratory of Crop Biology, National Engineering Laboratory for Efficient Utilization of Soil and Fertilizer Resources, Shandong Agricultural University, Taian, Shandong, China; 2 Shandong Rice Research Institute, Jinan, Shandong, China; 3 College of Mechanical and Electronic Engineering, Shandong Agricultural University, Taian, Shandong, China; The Ohio State University, United States of America

## Abstract

Appropriate tillage plays an important role in mitigating the emissions of greenhouse gases (GHG) in regions with higher crop yields, but the emission situations of some reduced tillage systems such as subsoiling, harrow tillage and rotary tillage are not comprehensively studied. The objective of this study was to evaluate the emission characteristics of GHG (CH_4_ and N_2_O) under four reduced tillage systems from October 2007 to August 2009 based on a 10-yr tillage experiment in the North China Plain, which included no-tillage (NT) and three reduced tillage systems of subsoil tillage (ST), harrow tillage (HT) and rotary tillage (RT), with the conventional tillage (CT) as the control. The soil under the five tillage systems was an absorption sink for CH_4_ and an emission source for N_2_O. The soil temperature positive impacted on the CH_4_ absorption by the soils of different tillage systems, while a significant negative correlation was observed between the absorption and soil moisture. The main driving factor for increased N_2_O emission was not the soil temperature but the soil moisture and the content of nitrate. In the two rotation cycle of wheat-maize system (10/2007–10/2008 and 10/2008–10/2009), averaged cumulative uptake fluxes of CH_4_ under CT, ST, HT, RT and NT systems were approximately 1.67, 1.72, 1.63, 1.77 and 1.17 t ha^−1^ year^−1^, respectively, and meanwhile, approximately 4.43, 4.38, 4.47, 4.30 and 4.61 t ha^−1^ year^−1^ of N_2_O were emitted from soil of these systems, respectively. Moreover, they also gained 33.73, 34.63, 32.62, 34.56 and 27.54 t ha^−1^ yields during two crop-rotation periods, respectively. Based on these comparisons, the rotary tillage and subsoiling mitigated the emissions of CH_4_ and N_2_O as well as improving crop productivity of a wheat-maize cropping system.

## Introduction

Methane (CH_4_) and nitrous oxide (N_2_O) play a key role in global climate change [1]. The global warming potential of these gases are respectively 25 and 298 times that of carbon dioxide (CO_2_) [2]; thus, the release of these gases is a crucial contributory factor to increasing loads of greenhouse gases (GHG). According to estimations of the IPCC [3], the fluxes of CH_4_ and N_2_O from agricultural sources account for 50% and 80% of the total emission of these gases, respectively. There have been many studies on CO_2_ emission in different ecosystems [4]–[6]; however, emissions of CH_4_ and N_2_O emission have been researched incompletely, especially in agricultural ecosystems [7], [8].

In general, appropriate soil tilling may reduce GHG emissions because the emissions from soil are strongly affected by tilling, results that have been found by many studies [8]–[10], most of which, have reported the emissions of CH_4_ and N_2_O under conventional tillage (CT) and no-tillage (NT) systems in different sites [5], [9]. However, the both generally revealed two extremes in maintenance the soil organic carbon stock and crop productivity, agricultural environment protection, the results in these aspects showed the regional character and sometimes they did not suit for agricultural sustainable development [8], [9], [11]. In which case, some reduced tillage systems such as subsoiling (ST), harrow tillage (HT) and rotary tillage (RT) have been introduced [11], and sometimes they as more important tillage practices combination with no tillage were used to in rotation-tillage systems, which changed some soil environment factors and crop yield [12]. Although these reduced systems were frequently used and developed rapidly due to they are not only advantageous to improve crop yield but also increase the utilization efficiency of soil, water and fertilizer [13], [14], the emissions of CH_4_ and N_2_O under these systems were remain unclear.

The production, consumption and transport of CH_4_ and N_2_O in soil are strongly influenced by some soil factors. Many studies demonstrated that CH_4_ uptake by soil is correlated with soil temperature [15], [16] and the N_2_O emission. The conditions of soil moisture and N concentration were also shown as two major driving factors of the emission of N_2_O: the emission generally peaked during N fertilizer application and irrigation [17], [18]. However, the effects of those factors on the emissions of CH_4_ and N_2_O under different tillage systems in the North China Plain are still not fully understood among available results.

Therefore, the aim of the present study was to quantify the emissions of CH_4_ and N_2_O under no tillage and three reduced tillage systems in the wheat-maize cropping system and to analyze the correlations between the two gas emissions and the soil temperature, moisture and nitrate content. The crop productivity of the wheat-maize cropping system during two crop-rotation periods was also analyzed.

## Materials and Methods

### Ethics Statement

This experiment was established in a long-term tillage and residue-management experiment site of Shandong Agricultural University. The farming operations of this experiment were similar to the rural farmers’ operations and did not involve endangered or protected species; the operations were approved by the State Key Laboratory of Crop Biology, Shandong Key Laboratory of Crop Biology and National Engineering Laboratory for Efficient Utilization of Soil and Fertilizer Resources, Shandong Agricultural University.

### Experimental Site

**Figure 1:**
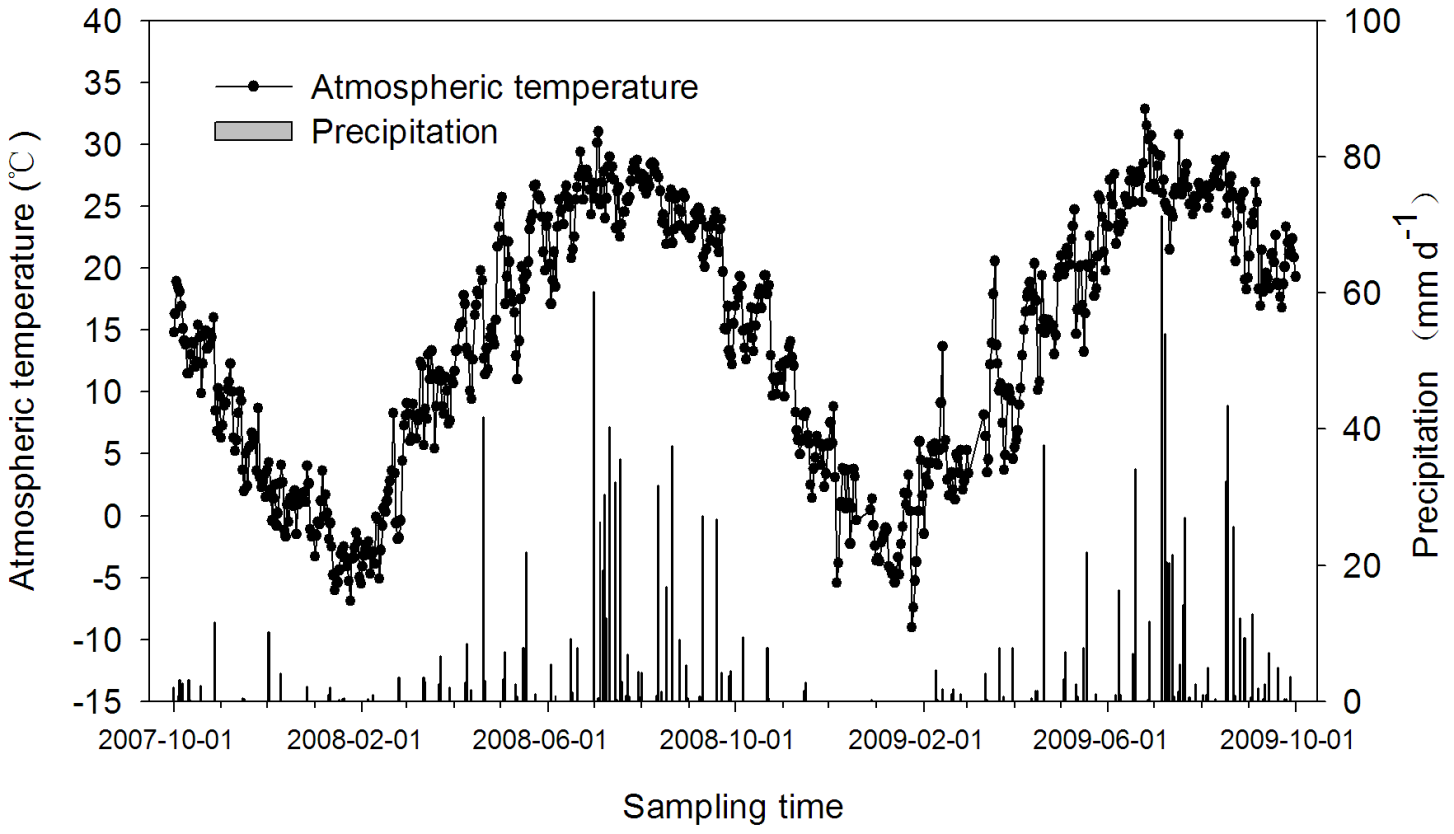
The atmospheric temperature and precipitation at the experimental site. The data were collected by the agricultural meteorological station approximately 500 m from the experiment field.

The study site was located at Tai’an (Northern China, 36°09′N, 117°09′E), which has the typical characteristics of the North China Plain. The average annual precipitation is 697 mm, and the average annual temperature is 13.0°C, with the minimum (−2.6°C) and maximum (26.4°C) monthly temperatures in January and July, respectively. The annual frost-free period is approximately 170–196 d in duration, and the annual sunlight time is 2627.1 hours. The soil is a loam with 40% sand, 44% silt, 16% clay. The characteristics of the surface soil (0–20 cm) were measured as follows: pH 6.8; soil bulk density 1.43g cm^−3^; soil organic matter 1.36%; soil total nitrogen 0.13%; and soil total phosphorous 0.13%. The meteorological data during the experiment is shown in Figure 1.

### Experimental Design

The study based on a 10-year tillage experiment, began in 2002, included no tillage (NT) and three reduced tillage systems involving subsoiling (ST), harrow tillage (HT), rotary tillage (RT), with the conventional tillage (CT) as the control. The treatments were arranged in a randomized block design with three replications. Each plot was 35 m long and 4 m wide.

The experimental site was cropped with a rotation of winter wheat (*Triticum aestivum* Linn.) and maize (*Zea mays L*.). Winter wheat (Jimai-22) was sown at a rate of 90 kg ha^−1^, on 12 October 2007 and 15 October 2008, and was harvested 6 June 2008 and 10 June 2009. The basal fertilizer was added before sowing and contained with 225 kg N ha^−1^, 150 kg P_2_O_5_ ha^−1^ and 105 kg K_2_O ha^−1^, and 100 kg N ha^−1^ was used as topdressing at the jointing stage with 160 mm of irrigation water. The maize was sown on 20 June 2008 and 22 June 2009, which included 66600 plants ha^−1^ and was harvested 8 October 2008 and 10 October 2009. For the maize, 120 kg N ha^−1^, 120 kg P_2_O_5_ ha^−1^ and 100 kg K_2_O ha^−1^ were used as a basal fertilizer, and 120 kg N ha^−1^ was used as topdressing at the jointing stage.

When the wheat and maize were harvested, the amount of residue retuned to each plot at an equal level according to the crop biomass and water content of residue, in order to ensure their amount and C content of the residue among treatment had no significant difference. The residue equal quality returned to the field, then pulverized using a residue chopper and mixed with the soil in the tillage operations. The operations of tillage and residue-management are shown in Table 1.

**Table 1:** The residue-management and tillage systems in the experimental plots.

Item	Tillage systems				
	CT	ST	HT	RT	NT
Wheat-residue					
Retention rate (t ha^−1^)	11.02	11.02	11.02	11.02	11.02
Residue-C (g kg^−1^)	51.27	51.27	51.27	51.27	51.27
Maize-residue					
Retention rate (t ha^−1^)	10.05	10.05	10.05	10.05	10.05
Residue-C (g kg^−1^)	52.15	52.15	52.15	52.15	52.15
Tillage					
Depth (cm)	25∼35	40∼45	12∼15	10∼15	–
Machine	moldboard	vibrating subsoil shovel	disc harrow	rototiller	–

### Gas Sampling and Analysis

The emission measurements of CH_4_ and N_2_O for each treatment were conducted using the static-chamber method [19]. According to some studies, there is optimal gas-sample collection duration in a day during which the sample can show the mean gas flux of the day [20]–[22]. Our previous study have showed that the ratios of the CH_4_ flux between 9 a.m. and 10 a.m. and N_2_O flux between 9 a.m. and 12 p.m. to the daily mean flux, respectively, verged 1 by the correction coefficient and regression analysis [23]. So, the CH_4_ and N_2_O were collected between 9 a.m. and 10 a.m. and between 9 a.m. and 12 p.m. respectively from October 2007 to August 2009 at approximately 1-month intervals [12], [23]. Both CH_4_ and N_2_O were sampled at 5 minutes, 20 minutes and 35 minutes using a needle tube. The diurnal variations of CH_4_ and N_2_O in this study were collected from 2^nd^ to 4^th^ of May at 2-hour interval in 2009. All gases samples were collected at same time in order to avoid the order difference of sampling time. The atmospheric temperature, the temperature in the static chamber, the soil surface temperature and the soil temperature at a depth of 5 cm were determined simultaneously.

The samples were measured using a Shimadzu GC-2010 gas chromatograph. CH_4_ was measured using a flame ionization detector with a stainless-steel chromatography column packed with a 5A molecular sieve (2 m long); the carrier gas was N_2_. The temperatures of the column, injector and detector were 80°C, 100°C and 200°C, respectively. The total flow of the carrier gas was 30 ml min^−1^, the H_2_ flow was 40 ml min^−1^, and the airflow was 400 ml min^−1^. N_2_O was measured using an electron-capture detector with a Porapak-Q chromatography column (4 m long); the carrier gas was also N_2_. The temperatures of the column, injector and detector were 45°C, 100°C and 300°C, respectively. The total flow of the carrier gas was 40 ml min^−1^, and the tail-blowing flow was 40 ml min^−1^. The gas fluctuations were calculated based on the gas-concentration change over time per unit area.

The emission fluxes of CH_4_ and N_2_O were calculated using the following formula [19]:
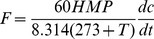
where *F* is the gas emission flux or uptake flux (µg m^−2^ hour^−1^), 60 is the conversion coefficient of minutes and hours, H is the height of the static chamber (m), M is the molar mass of gas (g mol^−1^), P is the atmospheric pressure (Pa), 8.314 is the ideal gas constant (J mol^−1 ^K^−1^), T is the average temperature in the static chamber (°C), and *dc/dt* is the slope of the line of the gas-concentration change over time.

The cumulative fluxes of CH_4_ and N_2_O were calculated by summing the products of the daily mean flux of two neighboring observations multiplying the days [6], [24], the calculation formula as follows:
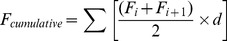


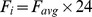
where, *F_cumulative_* is the cumulative flux of CH_4_ or N_2_O (t ha^−1^ year^−1^); *F_i_* is the daily flux of observation *i* (t ha^−1^ d^−1^); *F_i+1_* is the daily flux of observation *i+1* (t ha^−1^ d^−1^); *d* is the days; *F_avg_* is the mean flux of CH_4_ or N_2_O, which able to represent daily mean flux of CH_4_ or N_2_O according to the correction coefficient and regression analysis between the gas flux in observation duration and the daily total fluxes in diurnal variation.

### Soil Sampling and Analysis

The meteorological data during the experiment (10/2007–10/2009) were by the agricultural meteorological station approximately 500 m from the experiment field. We measured soil temperature at a depth of 5 cm and the soil moisture in the 0–20 cm soil layers using a WET Sensor (WET brand, made in the UK). Soil samples (0–10 cm, 10–20 cm and 20–30 cm) were collected at five random positions of each plot and air dried to a constant weight, triturated and passed through a 2 mm sieve after thorough incorporation; they were then used to determine NO_3_^–^N using the UV colorimetric method [25].

### Crop Yield

Winter wheat and maize were harvested at maturity, and the both harvest area was 9 m^2^ in the central area of each plot to exclude edge effects. After air-drying, the grains were separated from the plants and oven-dried at 65°C for 48 h, and the dry weight was determined.

### Statistical Analyses

The data were mapped using Sigma Plot 10.0, and all of the statistical analyses were performed using SPSS statistical software (SPSS Inc., Chicago, IL). The standard deviation (S.D.) and least significant difference (LSD) were calculated to compare the treatment means.

## Results

### Seasonal Variation of CH_4_ Uptake

**Figure 2:**
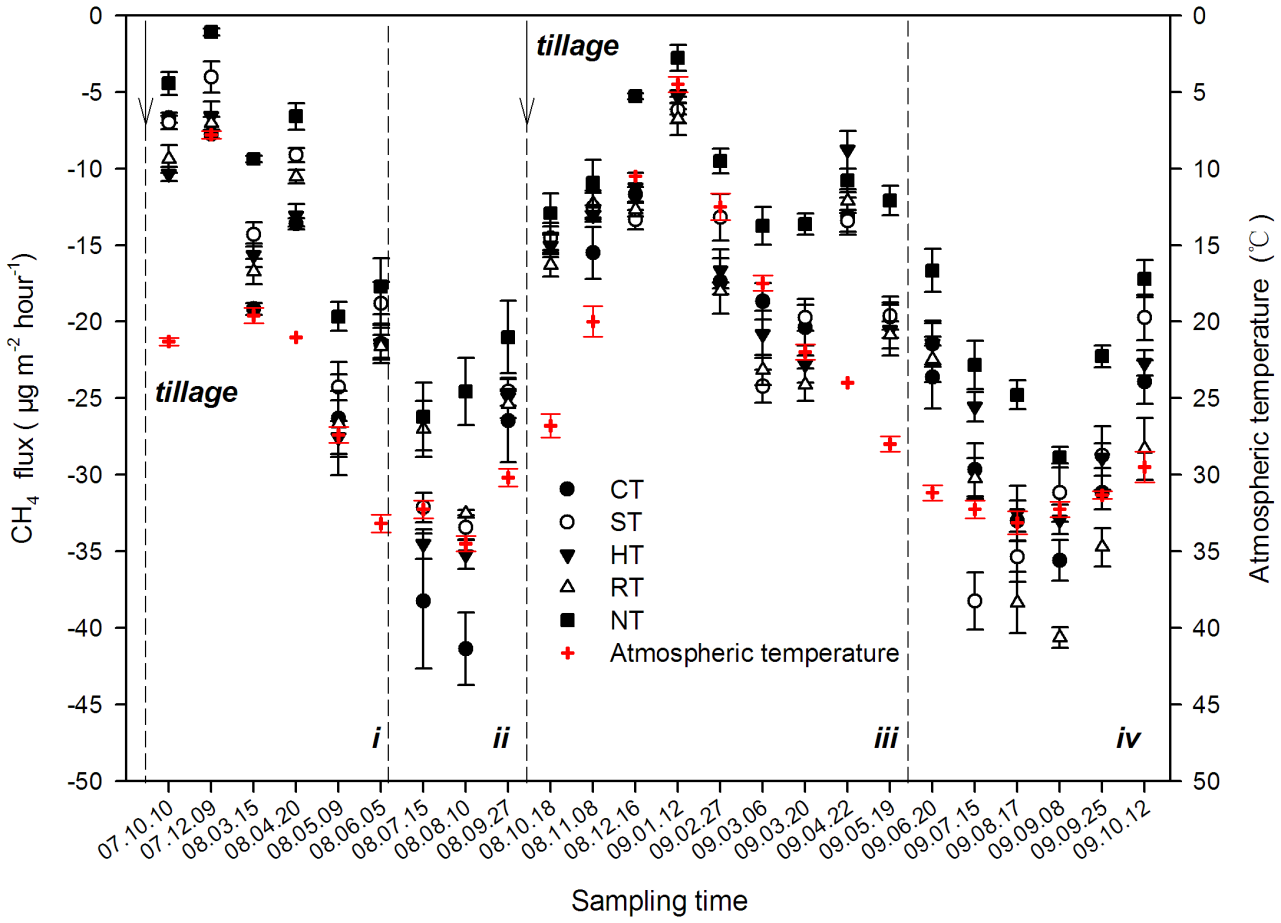
The seasonal characteristics of CH_4_ flux under the different tillage systems. i and iii were the periods of wheat growth in 2007–2008 and 2008–2009, respectively; ii and iv were the periods of maize growth in 2008 and 2009, respectively. The arrows indicate the time of tilling, and the dotted lines distinguish the different periods of crop growth. The data are means ± SD (n = 3).

The soil acted an absorption sink for CH_4_ in all tillage systems, which significantly varied by different soil tillage (Figure 2). The CH_4_ flux during the sampling period (10/2007∼10/2009) ranged from 6.31 to 41.36 µg m^−2^ h^−1^ under CT, from 4.03 to 33.45 µg m^−2^ h^−1^ under ST, from 5.30 to 35.21 µg m^−2^ h^−1^ under HT, from 6.78 to 32.54 µg m^−2^ h^−1^ under RT, and from 1.10 to 26.21 µg m^−2^ h^−1^ under NT. Moreover, the fluxes tended to respond to the change of atmospheric temperature during the sampling time (Figure 2), with higher uptake fluxes in the summer and lower in the winter in the different tillage systems.

### Seasonal Variation of N_2_O Emission

**Figure 3:**
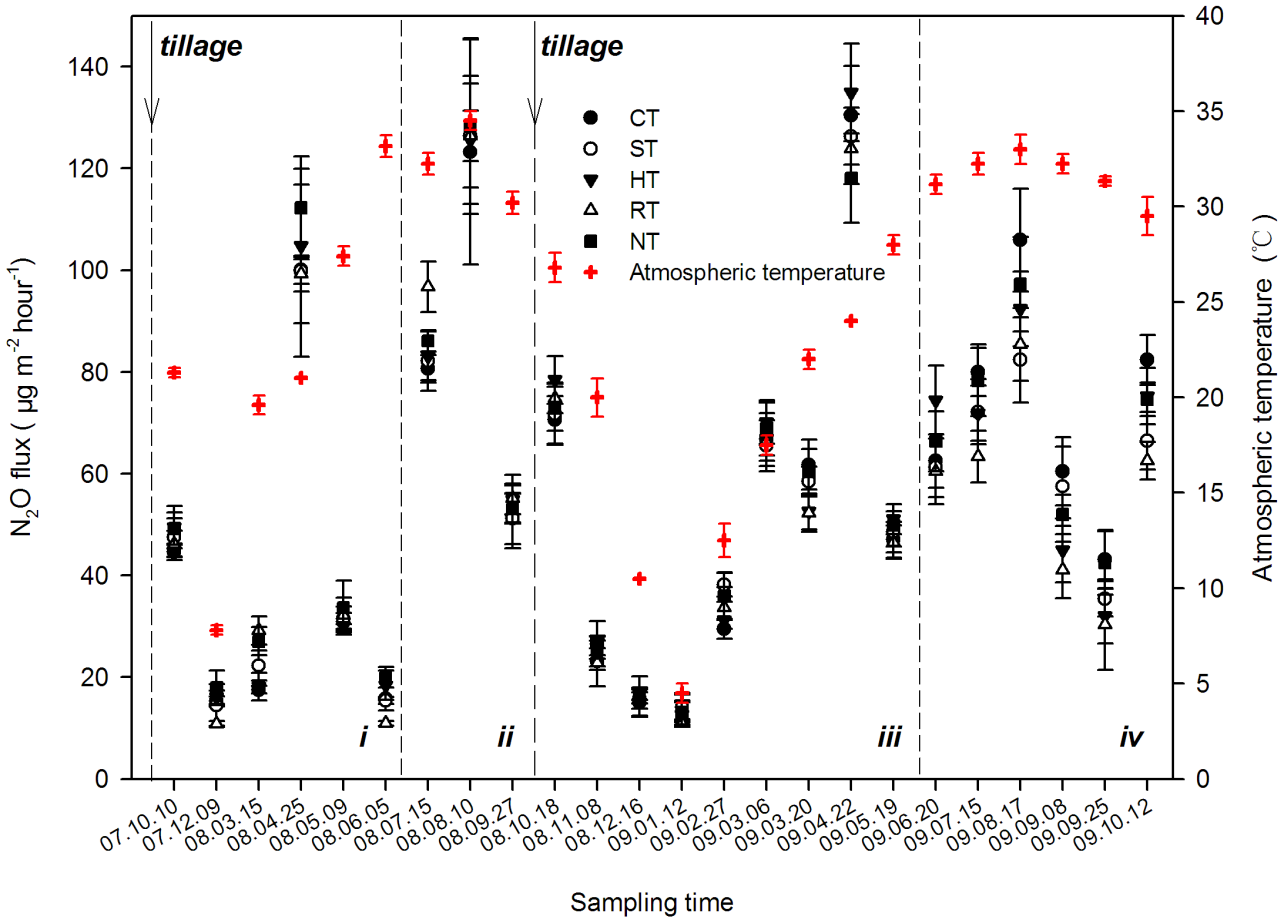
The seasonal characteristics of N_2_O flux under the different tillage systems. i and iii were the periods of wheat growth in 2007–2008 and 2008–2009, respectively; ii and iv were the periods of maize growth in 2008 and 2009, respectively. The arrows indicate the time of tilling, and the dotted lines distinguish the different periods of crop growth. The data are means ± SD (n = 3).

The emitting of N_2_O from the soil was observed under the different treatments (Figure 3), but the differences were small among the treatments in the same sampling time. However, the peak N_2_O emission coincided with the irrigation and fertilization periods, which had the highest emission fluxes of all of the periods (*P*<0.01), and the flux did not accord with the trend of atmospheric temperature. Meanwhile, the flux in all of the samples of N_2_O ranged from 14.07 to 130.39 µg m^−2^ h^−1^ under CT, from 14.20 to 126.43 µg m^−2^ h^−1^ under ST, from 12.68 to 134.93 µg m^−2^ h^−1^ under HT, from 10.81 to 126.42 µg m^−2^ h^−1^ under RT, and from 13.04 to 128.29 µg m^−2^ h^−1^ under NT.

### Diurnal Variations of CH_4_ and N_2_O Under CT and NT

**Figure 4:**
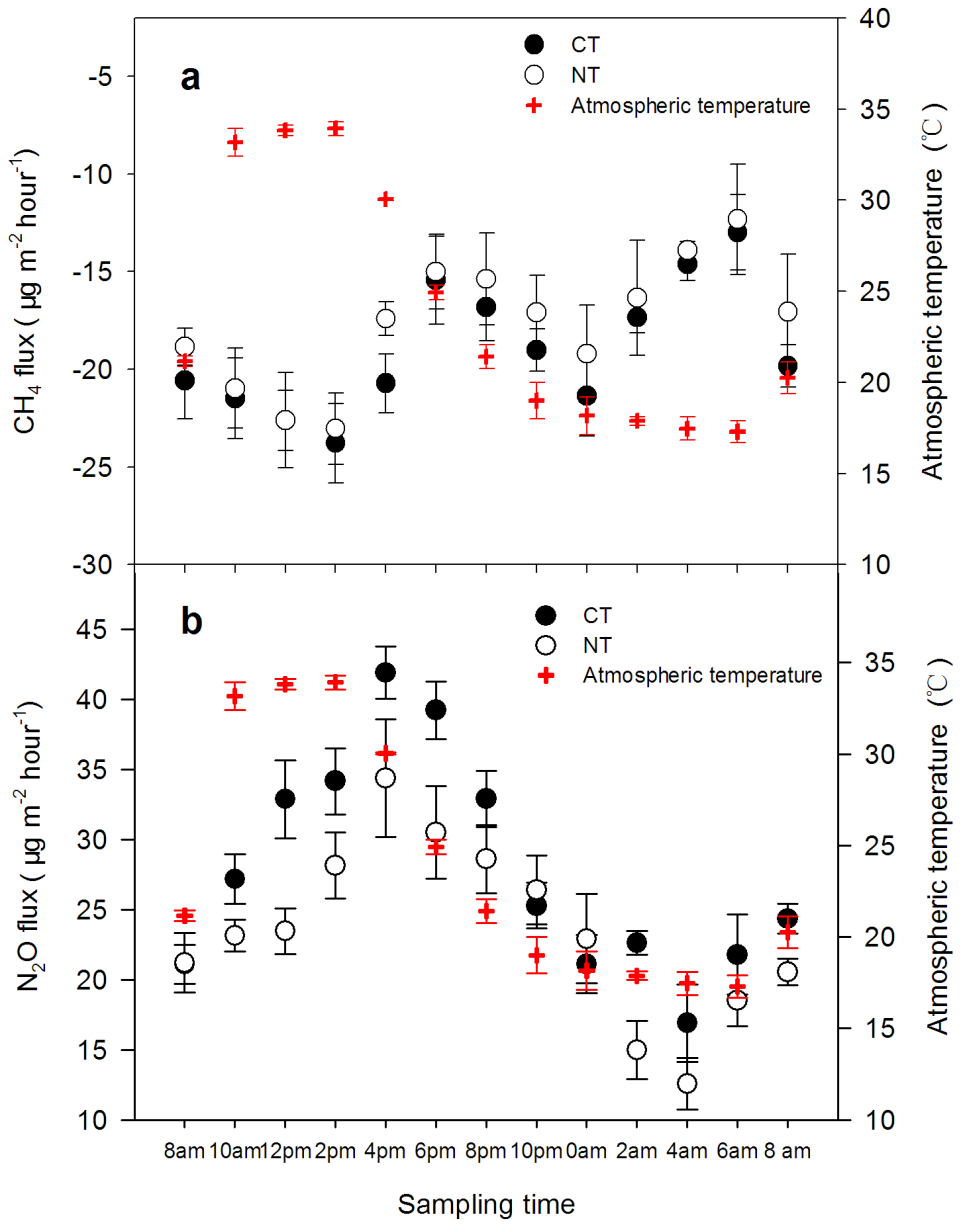
The diurnal flux variations of CH_4_ uptake (a) and N_2_O emission (b) in the CT and NT treatments. The data were collected from 2^nd^ to 4^th^ of May at 2-hour intervals in 2009. The data are means ± SD (n = 3).

The diurnal flux variations of CH_4_ uptake and N_2_O emission significantly differed between the CT and NT treatments (Figure 4). In both of the treatments, the CH_4_ uptake exhibited the lowest and highest fluxes at 6 a.m. and 2 p.m., respectively, but the lowest and highest values of CH_4_ absorption were 12.99 and 23.77 µg m^−2^ h^−1^, respectively, under CT and 12.23 and 23.03 µg m^−2^ h^−1^, respectively, under NT (Figure 4a). The emission troughs and peaks of N_2_O under CT and NT were observed at 4 a.m. and 4 p.m., respectively, with flux values of 16.90 and 41.89 µg m^−2^ h^−1^ under CT and 13.57 and 34.38 µg m^−2^ h^−1^ under NT, respectively (Figure 4b).

### Cumulative Emissions of CH_4_ and N_2_O

The cumulative fluxes of CH_4_ and N_2_O under the different tillage systems in the two rotation cycle of wheat-maize systems were shown in Table 2. The highest cumulative uptake flux of CH_4_ presented in the RT treatment with 1.85 t ha^−1 ^year^−1 ^in the first rotation cycle of wheat-maize system (10/2007–10/2008), which was higher 8.3%, 13.7%, 22.5% and 60.9% than CT, ST, HT and NT treatments, respectively. But in the second rotation cycle (10/2008–10/2009), the highest one was the ST treatment. The order of total cumulative uptake fluxes of CH_4_ under five tillage systems in two years was RT>ST>CT>HT>NT. The cumulative emission flux of N_2_O ranged from 4.18 to 4.63 t ha^−1 ^year^−1 ^in the first year and from 4.15 to 4.67 t ha^−1 ^year^−1 ^in the second year, the total cumulative emission flux of N_2_O ordered of NT>HT>CT>ST>RT, the flux under NT was only higher 7.2% than that of under RT.

**Table 2:** The cumulative emissions of CH_4_ and N_2_O under the different tillage systems.

Item	Cumulative flux (t ha^−1 ^year^−1^)				
	CT	ST	HT	RT	NT
The first rotation cycle of wheat-maize system (10/2007–10/2008)					
Cumulative uptake flux of CH_4_	1.71b	1.63c	1.51d	1.85a	1.15e
Cumulative emission flux of N_2_O	4.18d	4.30c	4.37c	4.45b	4.64a
The second rotation cycle of wheat-maize system (10/2008–10/2009)					
Cumulative uptake flux of CH_4_	1.63d	1.81a	1.74b	1.67c	1.20e
Cumulative emission flux of N_2_O	4.67a	4.46c	4.57b	4.15d	4.57b
The total cumulative flux in the two years					
CH_4_	3.34	3.43	3.25	3.53	2.34
N_2_O	8.85	8.76	8.94	8.60	9.22

Different small letters in the same line indicate *P*<0.05. n = 3.

### Seasonal Variations of the Soil Temperature, Moisture and NO_3_^–^N

**Figure 5:**
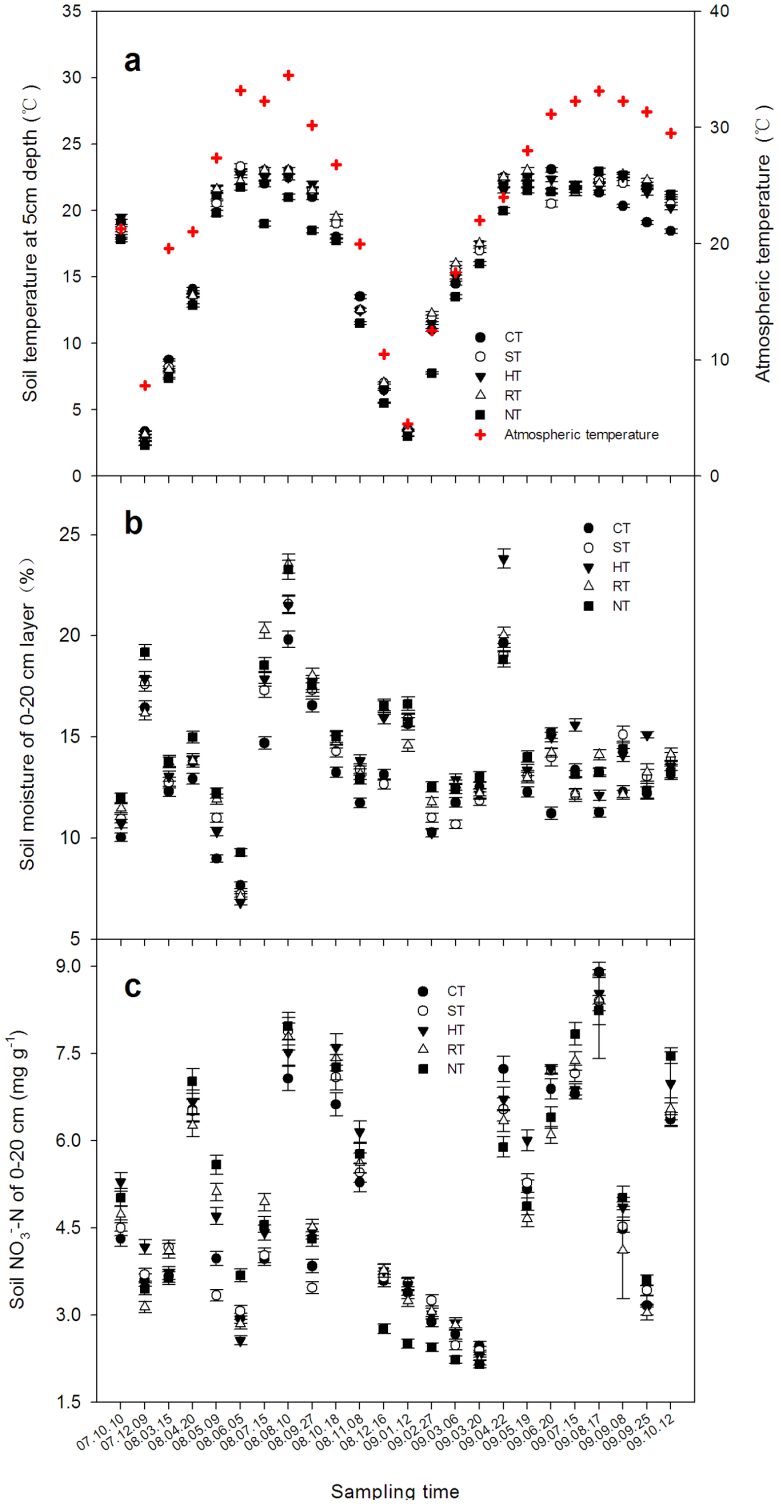
The seasonal variations of the soil temperature at a depth of 5 cm (a), soil moisture at 0–20 cm (b), and soil NO_3_^–^N content at 0–20 cm (c) under the different tillage systems. The data are means ± SD (n = 3).

A significant difference of the soil temperature at 5 cm depth was measured in the different periods. The changes under the different tillage systems were related to the atmospheric temperature (Figure 5a). The averaged soil temperature in all of the periods under RT was higher than under the other tillage methods. The soil moisture of the 0–20 cm layer varied among the different treatments and was related to precipitation or irrigation (Figure 5b). The averaged moisture level of the 0–20 cm layer was highest in the NT treatment. Similarly, higher nitrate contents were measured under the HT, NT, RT and ST treatments (Figure 5c); the levels in the NT, RT and ST treatments were higher than in the CT treatment by 4.21%, 2.42% and 1.40%, respectively.

### Regression Analysis between CH_4_, N_2_O and Soil Factors

**Figure 6:**
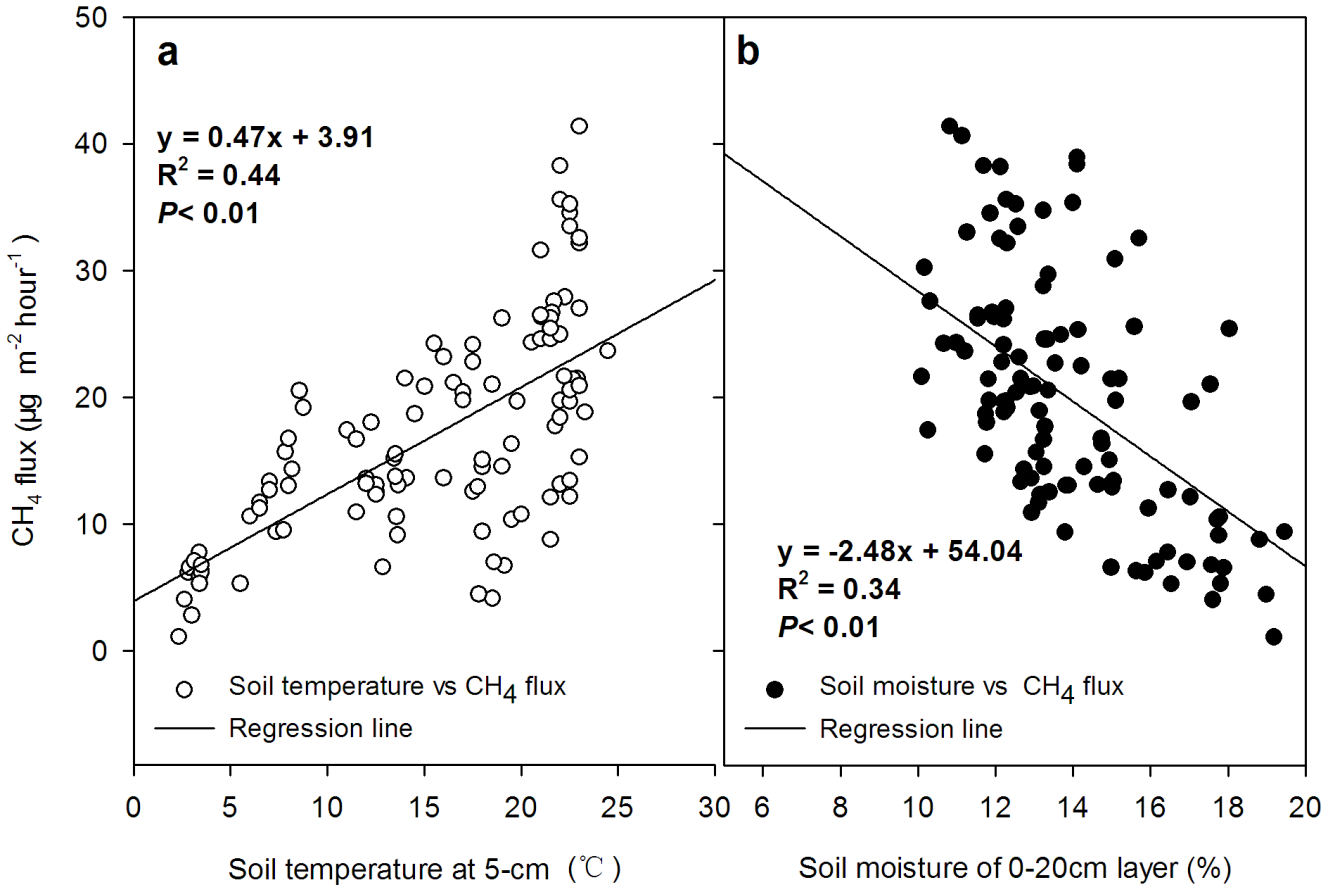
The regression analysis of the CH_4_ flux and soil temperature at 5-cm depth (a, n = 105) and soil moisture of the 0–20 cm layer (b, n = 105).

The absorption of CH_4_ by the soil in different tillage systems was strongly affected by the soil temperature and soil moisture, and the uptake flux showed a positive correlation with the soil temperature (R^2^ = 0.44, *P*<0.01; Figure 6a), and a negative correlation was observed with the soil moisture (R^2^ = 0.36, *P*<0.01; Figure 6b).

**Figure 7:**
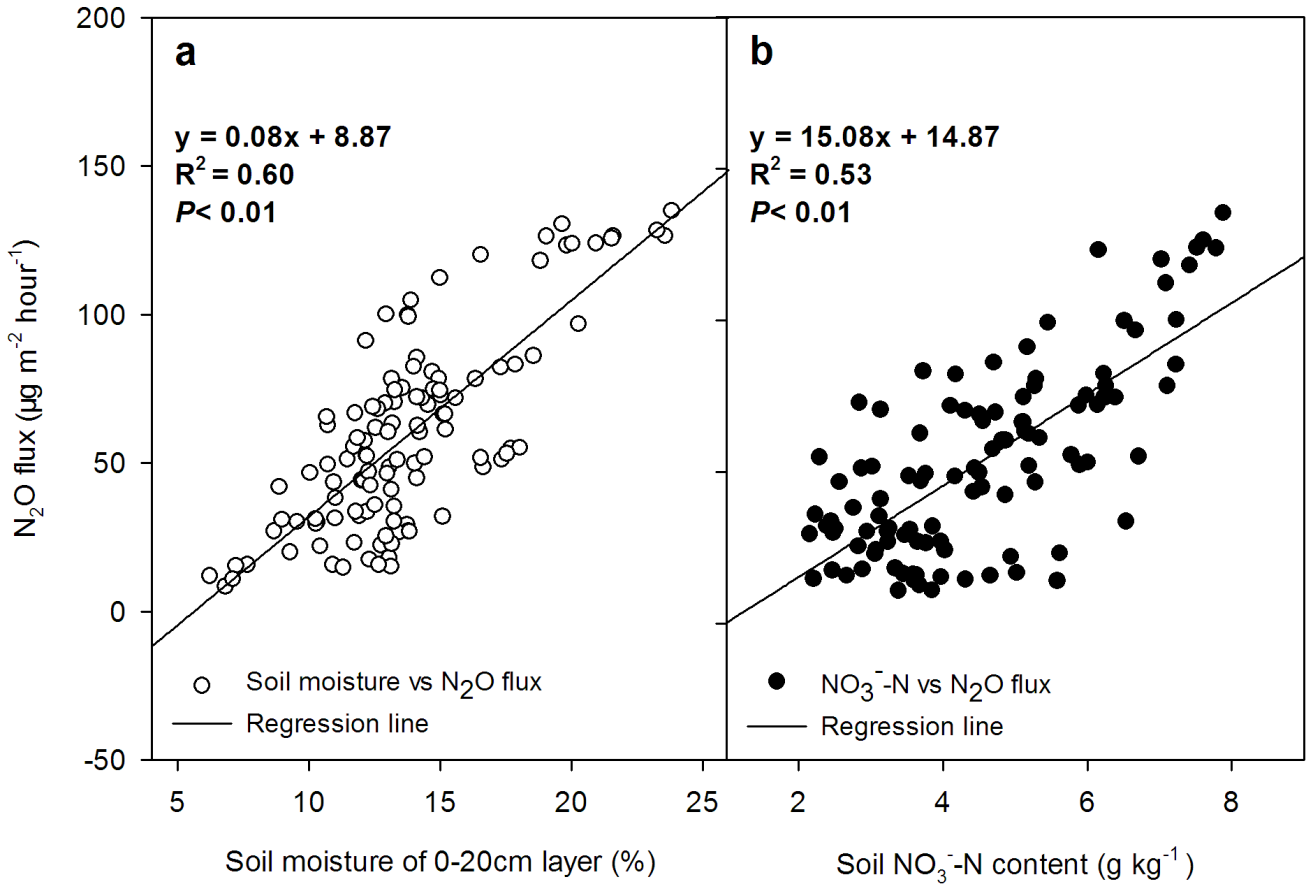
The regression analysis of the N_2_O flux and soil moisture of the 0–20 cm layer (a, n = 105) and the soil NO_3_^–^N of the 0–20 cm layer (b, n = 105).

The N_2_O emission flux and soil temperature were not significantly correlated in this study. However, the N_2_O emission flux was significantly related to the soil moisture (R^2^ = 0.63, *P*<0.01; Figure 7a) and the content of nitrate (R^2^ = 0.50, *P*<0.01; Figure 7b), which promoted the N_2_O emission.

### Crop Yields

The crop productivity of the wheat-maize rotation in the two years differed among the tillage systems (Table 3). The highest total productivity in two crop-rotation periods were measured in the ST treatment, with 34.63 t ha^−1^, which was higher 2%, 6.2%, 0.2% and 25.7% than in the CT, HT, RT and NT treatment. The NT system showed the lowest productivity, only producing 27.54 t ha^−1^ yield in two crop-rotation periods.

**Table 3:** The crop productivity in the different tillage systems.

Wheat-maize cropping-rotation system	Crop yield (t ha^−1^)				
	CT	ST	HT	RT	NT
The first rotation cycle					
Wheat (10/2007∼06/2008)	5.90 d	6.18 c	6.58 a	6.35 b	4.65 e
Maize (06/2008∼10/2008)	12.19 a	10.98 b	10.10 c	9.92 d	8.83 e
The second rotation cycle					
Wheat (10/2008∼06/2009)	5.85 c	6.90 a	6.17 b	6.06 b	4.95 d
Maize yield (06/2009∼10/2009)	10.03 c	10.58 b	9.76 d	12.23 a	9.11 e
Total productivity	33.95 b	34.63 a	32.62 c	34.56 a	27.54 d

Different small letters in the same line indicate *P*<0.05. n = 3.

## Discussion

### Effects of Soil Factors on CH_4_ Uptake and N_2_O Emission

In general, the emissions of CH_4_ and N_2_O in different seasons are affected by the soil temperature [26], [27]. In this study, the CH_4_ uptake and N_2_O emission were lower in winter and higher in summer (Figures 2 and 3) and significantly related with the change of the soil temperature (Figures 2 and 6a). Similar results have been indicated in previous studies [28], [29]. However, the CH_4_ uptake flux usually decreased with the irrigation (Figures 3 and 6b). Many studies reported that soil moisture was a limiting factor for CH_4_ absorption by the soil [16], [30], leading to reduced rates of gas and O_2_ diffusion [17].

Sometimes, the seasonal characteristics of N_2_O emission exhibit varied trends in different sites, generally affected by temperature, N fertilization or irrigation in a significant linear relationship [27], [31], and higher NO_3_-N concentrations in wet soils promote the activity of nitrification and denitrification [18]. The higher moisture of the soil in this study promoted N_2_O emission (Figures 5b and 7a), and the emission also increased with N fertilizer application (Figures 5c and 7b). In addition, there was no significant correlation between the N_2_O emission and soil temperature in the seasonal variation in this study, similar results also found by other studies [32].

### Tillage Effect on CH_4_ Uptake and N_2_O Emission

The fluxes of CH_4_ uptake and N_2_O emission were related to some soil factors (Figures 6 and 7), and these related soil factors general varied by the different tillage systems (Figure 5), because the changed soil structure and microflora by tillage would further drive emissions of the N_2_O and CH_4_ from soil [8], [15]. For example, a soil with better permeability was a larger absorption sink of CH_4_ [5]. In this study, the highest uptake flux of CH_4_ was observed in the RT treatment (Table 2), which also contained the highest averaged soil temperature (Figure 5a). The regression analysis showed that there was a significant positive correlation between CH_4_ uptake and soil temperature (Figure 6a). In contrast, under the five tillage systems used in this study, the lowest flux of CH_4_ uptake was observed under NT (Figure 2), which was consistent with many previous studies [13], [14]. The highest moisture and the lowest temperature conditions were also found in the soil of the NT treatment (Figures 5a and 5b). Some previous studies have indicated that the excessive wet condition of soil generally led to compaction of the soil surface without tillage, which blocked CH_4_ from entering into soil for oxidation in dry-land farming systems [32]–[35]. However, others opposite results also indicated that NT could increase CH_4_ oxidation because the reduced disturbance of soil could increase the activity of methane-oxidizing bacteria [36], [37], while disturbance may negatively affect CH_4_ uptake by the soil [38], but sometimes this effect from disturbance is small and can largely be ignored [9], [38]. Because the variations of these factors are important driving factors for soil microflora and also responded sensitive by the changed soil structure by tillage.

Similarly, the HT treatment, which had the highest emission of N_2_O from the soil because the content of NO_3_-N was the highest compared with the other treatments (Figures 5c and 7b). The emission flux of N_2_O usually peaked after the stage of N-fertilizer application or irrigation [10], and the highest flux was observed in reduced systems (HT treatment). In some cases, there was a risk of increasing the emission of N_2_O under NT or reduced tillage [9]. These methods mostly produced decreased N_2_O emission relative to that reported by many previous studies [39], [40] because little N_2_O was generally produced with a better mixture of soil and residue, and the predominant form of nitrogen was NO_3_-N or NH_4_-N [14].

### Tillage Effect on Crop Yield

Little information was reported on reduced tillage systems effect on crop yields. Sometimes, subsoiling was regarded an effective method to increase wheat production [41]–[43]. In this study, the highest crop productivity of two rotation periods was shown under the ST and RT systems (Table 3). However, the NT system in this study had the lowest crop total yield, and a similar trends were reported by other studies [5], [10]. However, the yields under NT showed dissimilar results in different sites, in which generally reported with the NT increase crop yields compared to conventional tillage [44], [45]. Furthermore, relative to other tillage systems, no-tillage grain yields and profits are often dramatically lower during the first few years of adoption. In fact, no tillage have not been widely applied in the world or at least China, because there are actual or perceived problems in crop grown using the NT, it may have limited adoption by growers. Some specific problems include lower early-season soil temperatures, reduced seed germination and emergence, below-optimal plant populations, poorer weed control, delayed plant development and maturity, increased grain moisture content, and lower grain yield potential [46]–[51]. Yet no tillage system effects on yield are highly dependent upon soil type, drainage, climate/latitude, and crop rotation [52].

## Conclusions

The fluxes of CH_4_ and N_2_O were significantly affected by no tillage and reduced tillage systems, which also impacted soil factors, such as the soil temperature, moisture and nitrate content. These factors were related with the changes of CH_4_ and N_2_O fluxes. No-tillage did not reveal a better result for mitigating emissions of CH_4_ and N_2_O, as well as maintaining a high level of crop yield in this cropping rotation system. Comparison to the conventional tillage and no tillage, under the reduced tillage systems such as subsoiling and rotary tillage, may mitigate emissions of CH_4_ and N_2_O, and also gain a high level of crop productivity in the wheat-maize cropping rotation system. Sometimes, the both also as an important rotation tillage systems was applied in this region, in particular the subsoiling. The results also provide information on optional tillage in rotation tillage systems for mitigating GHG emissions and improving crop yield.
